# On the Non-trivial Origin of Atomic-Scale Patterns in Friction Force Microscopy

**DOI:** 10.1007/s11249-018-1127-6

**Published:** 2018-12-31

**Authors:** Dirk W. van Baarle, Sergey Yu. Krylov, M. E. Stefan Beck, Joost W. M. Frenken

**Affiliations:** 1 Advanced Research Center for Nanolithography, Science Park 106, 1098 XG Amsterdam, The Netherlands; 2 Huygens - Kamerlingh Onnes Laboratory, Leiden University, P.O. Box 9504, 2300 RA Leiden, The Netherlands; 3 Institute of Physical Chemistry and Electrochemistry, Russian Academy of Sciences, Moscow, Russia 119071; 4 Institute of Physics, University of Amsterdam, Science Park 904, 1098 XH Amsterdam, The Netherlands

**Keywords:** Friction force microscopy, Atomic-scale friction, Energy dissipation, Stick-slip motion

## Abstract

Friction between two surfaces is due to nano- and micro-asperities at the interface that establish true contact and are responsible for the energy dissipation. To understand the friction mechanism, often single-asperity model experiments are conducted in atomic-force microscopes. Here, we show that the conventional interpretation of the typical results of such experiments, based on a simple mass-spring model, hides a fundamental contradiction. Via an estimate of the order of magnitude of the dissipative forces required to produce atomic-scale patterns in the stick-slip motion of a frictional nano-contact, we find that the energy dissipation must be dominated by a very small, highly dynamic mass at the very end of the asperity. Our conclusion casts new light on the behavior of sliding surfaces and invites us to speculate about new ways to control friction by manipulation of the contact geometry.

## Introduction

Friction, the familiar force experienced both on the macroscopic scale in everyday life and on the scale of atoms and molecules, is directly related to the dissipation of energy. One may hope that a full understanding of the phenomenon will ultimately result in complete control of friction, which can be employed, e.g., to eliminate undesired forms of friction and associated wear in engines and other mechanical applications. In this context, special attention is given to the origin of friction [1–3 and refs. therein].

**Fig. 1:**
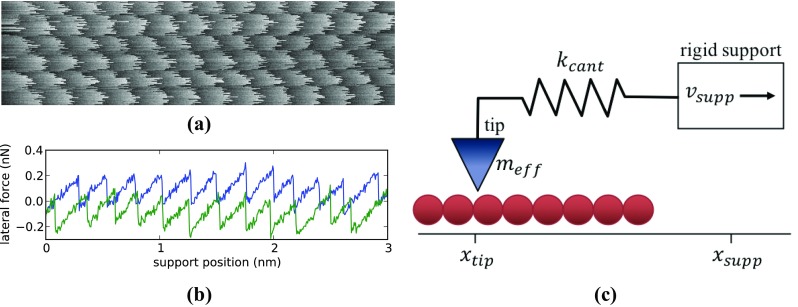
**a** Typical lateral force map 3 nm × 0.8 nm, obtained by friction force microscopy. In each line, the silicon tip moves from left to right over a graphite substrate and the gray scale indicates the strength of the opposing lateral force. **b** Lateral force variation during an individual sweep of the tip from left to right (blue curve; center line in panel **a**) and from right to left (green). **c** Traditional schematic view of the FFM-experiment; a rigid support (white rectangle) is moving at constant velocity and drags the tip (blue triangle) via a spring over the atomically corrugated substrate (red spheres). The extension of the spring is a direct measure of the lateral force experienced by the tip. Data in panels (**a**) and (**b**) with courtesy of Prof. A. Schirmeisen (Justus-Liebig University, Gießen, Germany); further details on these measurements can be found in [4]

One of the key instruments in the experimental investigation of friction at the nano-scale is the friction force microscope (FFM) [2], in which the tip–surface contact serves as a model for a single-asperity contact. Two-dimensional maps of the lateral force experienced by the tip in an FFM-experiment often display a clear, atomic-scale periodicity, as is illustrated in Fig. 1a and b. Such patterns reflect the stick-slip (SS) motion that the tip executes between nearby minima in the corrugated tip–substrate interaction potential.

Here we use the mere observation of such SS-behavior to draw conclusions about both the kinetic energy and the energy dissipation rate that are in play during a typical atomic slip event in an FFM. We demonstrate that the amount of energy one should reasonably expect to be dissipated by the atomic-size contact is some six orders of magnitude lower than what is typically assumed. This completely invalidates the traditional interpretation of the observed slip dynamics. Instead, we propose a more sophisticated dissipation mechanism that involves the dynamics of a tiny mass at the very end of the tip apex. Our mechanism is consistent with the experimental observations. In addition, it provides more general insight in sliding and friction and may open the possibility to predict the friction behavior of both typical and non-typical contact geometries.

## Basic Considerations

We start by inspecting typical SS-motion (Fig. 1b) in more detail. It is characterized by an initial ‘stick’ part, in which a mechanical force is gradually building up between the stationary tip and a rigid support that moves at constant velocity, and a rapid ‘slip’ event, during which the invested potential energy is transferred into kinetic energy and the tip translates over one lattice spacing. This motion is interpreted in the context of the well-known Prandtl–Tomlinson model [5, 6] as the natural behavior of a mass-spring system that is pulled over a corrugated surface, as shown schematically in Fig. 1c. Mathematically, it is described by a Langevin equation for the *x*-direction

1 $${m_{{\text{eff}}}}{\ddot {x}_{{\text{tip}}}}= - {\left. {\frac{{\partial {V_{{\text{int}}}}\left( {x,y} \right)}}{{\partial x}}} \right|_{\left( {x,y} \right)=\left( {{x_{{\text{tip}}}},{y_{{\text{tip}}}}} \right)}} - {k_{{\text{cant}}}}\left( {{x_{{\text{tip}}}} - {x_{{\text{supp}}}}} \right)+\xi - \gamma {\dot {x}_{{\text{tip}}}},$$

and an equivalent equation for the *y*-direction, where $\left( {{x_{{\text{tip}}}},{y_{{\text{tip}}}}} \right)$ is the position of the tip in the surface plane. ${m_{{\text{eff}}}}$ represents the effective mass of the tip and the atomically corrugated tip–substrate interaction potential is given by ${V_{{\text{int}}}}$. The spring force resulting from the difference between the positions of support and tip is governed by an effective spring constant ${k_{{\text{cant}}}}$, which is a combination of the stiffness of the cantilever of the FFM and the stiffness of the tip–surface contact and can be deduced directly from the slope of the measured lateral force trace in Fig. 1b [7]. In addition to the two static forces, one due to the interaction with the substrate and the other due to the spring, the tip also experiences two dynamic forces. The first, $\xi$, is due to thermal noise, while the second, $\gamma {\dot {x}_{tip}}$, is the velocity-dependent dissipation. For the present study, the dissipation term establishes the most important term in the equation; it is through this term that friction is introduced. Note that the values of the dissipation rate $\gamma$ and the amplitude of the noise term $\xi$ cannot be ‘chosen’ independently, as they are intimately connected via the fluctuation–dissipation theorem.

Most numerical calculations that are used to reproduce FFM-experiments apply the ‘recipe’ of Eq. 1. Typically, the effective ‘tip’ mass is assumed to be in the order of 10^−11^ kg, which corresponds to the combination of the tip and a sizable fraction of the cantilever that moves together with the tip. The dissipation rate $\gamma$ is almost always used as the fitting parameter to reproduce the experimental data, which results in a typical value of 10^−6^ kg s^−1^, corresponding to tip motion that is in the order of critically damped [3, 4, 8–18]. Although this approach produces atomic patterns, it does not lead to a physical understanding of the resulting $\gamma$-values, for example in terms of the microscopic dissipation mechanism.

## Dissipation as a Sum of Atomic Contributions

In this study, we approach the subject from the other side, by first considering the dissipation rates that are familiar on the scale of single atoms. There is ample evidence from the fields of the vibrations and diffusion of atoms and molecules on surfaces, e.g., through a variety of calculations and through the observation of a modest percentage of long jumps of diffusing atoms, that the motion on this small scale is close to critically damped [19–25]. In other words, the typical timescale on which an atom, intimately adsorbed on a surface, can get rid of its excess momentum is in the order of half its own natural vibrational period on that surface, which we express as

2 $$\frac{{{\gamma _{{\text{at}}}}}}{{{m_{{\text{at}}}}}} \cong 2\sqrt {\frac{{{k_{{\text{at}}}}}}{{{m_{{\text{at}}}}}}} .$$

Here, ${m_{{\text{at}}}}$ is the mass of an atom, ${k_{{\text{at}}}}$ is the typical spring coefficient, binding each atom to its equilibrium position, and ${\gamma _{{\text{at}}}}$ is the atomic dissipation rate. In order to turn this into a simple, order-of-magnitude estimate for the friction that we should expect between a sharp tip and a surface, we assume that an atomically sharp tip would experience the same friction force (momentum dissipation rate) as the final tip apex atom would feel on that surface, without the rest of the tip connected to it. We further assume that the friction force on a blunter tip is simply proportional to the number of atoms ${N_{{\text{cont}}}}$ by which the tip is in contact with the surface.

3 $${F_{{\text{diss}}}}= - \gamma {\dot {x}_{{\text{tip}}}}= - {N_{{\text{cont}}}}{\gamma _{{\text{at}}}}{\dot {x}_{{\text{tip}}}}.$$

Note that Eq. 3 does not imply that dissipation would be restricted to the contact atoms. It does, however, pay tribute to the fact that the mechanical excitations in the sliding interface that lead to friction certainly originate from the contact, even when dissipation of these excitations occurs further away in the substrate and the tip body. Thereby, it is the contact area, i.e., the number of atoms in contact ${N_{{\text{cont}}}}$, that determines how much mechanical energy is temporarily stored in the contact and subsequently dissipated in a slip event. In Appendix A, we provide a further justification for the assumed proportionality between the friction force and ${N_{{\text{cont}}}}$. By relating the sliding friction force directly to the microscopic, true area of contact [26], to the actual, interfacial velocity, and to the atomic-scale dissipation rate, we propose to put the phenomenological Amontons–Coulomb law on a truly fundamental footing for the case of wearless friction.

A typical value for ${N_{{\text{cont}}}}$ for FFM and contact-AFM experiments is 10. For a typical atomic mass number of 74 amu (tungsten) and a typical atomic or short-wavelength lattice vibration frequency of 10^12^ Hz, this leads to a dissipation rate of the contact of 10^−11^ kg s^−1^, which is some five orders of magnitude lower than the values required to reproduce atomic patterns in FFM-calculations. The two assumptions that have gone into Eq. 3 may be crude, but a more refined description will not remove the blatant discrepancy.

At this point, we draw the conclusion that the values adopted for the dissipation parameter in most FFM-calculations are unphysically high. Correspondingly, calculations within the same model for realistic $\gamma$-values would result in highly underdamped motion and could never reproduce a regular SS-pattern with lattice periodicity.

## Strong Dissipation Implies Ultralow Mass

In order to remedy the phenomenal discrepancy encountered above, we return to Eq. 1. The only remaining element that can be completely incorrect in the employed, typical estimates is the assumed value of the effective mass ${m_{{\text{eff}}}}$ [18]. What does this mass stand for and how does its value affect the friction behavior? The effective mass represents the number of dynamic atoms ${N_{{\text{dyn}}}}$ that effectively move at the same velocity as the contact and it is usually associated with the entire tip plus a part of the FFM-cantilever, hence its large, typical value of 10^− 11^ kg. Since the maximum velocity ${\dot {x}_{{\text{tip}}}}$ that the tip apex can acquire during a slip event in the absence of damping (cf. Equation 1) is inversely proportional to the square root of the effective mass, this mass also influences the maximum friction force ${F_{{\text{diss}}}}$ that the tip apex can experience in this motion (cf. Equation 3). We should thus expect a simple scaling between the maximum dissipative force and the effective mass of the type $F_{{{\text{diss}}}}^{{{\text{max}}}} \propto 1/\sqrt {{m_{{\text{eff}}}}}$. So, the required increase in the friction force by five orders of magnitude translates directly into a reduction in the effective mass by at least ten orders of magnitude with respect to the value typically assumed. Obviously, we can no longer associate this with the entire tip and part of the FFM-cantilever, but see that this small mass can only stand for a very small portion of the tip, namely its very apex. We will refer to this tiny mass as the dynamic mass ${m_{{\text{dyn}}}}$ and will be forced to describe its dynamics and that of the rest of the tip and cantilever with separate equations of motion (see below).

It is instructive to cast the above argument about the dynamic mass ${m_{{\text{dyn}}}}$ in terms of the two typical timescales involved. One is introduced by the timescale of a slip event, which we equate, here, to half the vibrational period of the slipping tip apex, ${t_{{\text{slip}}}}=\frac{1}{2}\sqrt {{m_{{\text{dyn}}}}/{k_{{\text{dyn}}}}}$. Here, ${k_{{\text{dyn}}}}$ stands for the effective spring coefficient by which the dynamic mass is connected to the rest of the system, which is the stiffness of the tip apex itself. The other is the time required to dissipate all kinetic energy via the ${N_{{\text{cont}}}}$ contact atoms. It is directly related to the dissipation rate $\gamma$ in Eq. 3, through ${t_{{\text{diss}}}}\,=\,~{m_{{\text{dyn}}}}/\gamma \,=\,{m_{{\text{dyn}}}}/{N_{{\text{cont}}}}{\gamma _{{\text{at}}}}$, which we can rewrite to ${t_{{\text{diss}}}}=\frac{1}{2}{m_{{\text{dyn}}}}/{N_{{\text{cont}}}}\sqrt {{k_{{\text{at}}}}{m_{{\text{at}}}}}$ using Eq. 2. In order to obtain patterns with atomic periodicity, we require the dynamic part of the system to be at least critically damped, i.e., ${t_{{\text{diss}}}} \leqslant {t_{{\text{slip}}}}$. We can now write this critical-damping condition in terms of the spring coefficients and the numbers of atoms involved:

4 $${N_{{\text{dyn}}}} \equiv \frac{{{m_{{\text{dyn}}}}}}{{{m_{{\text{at}}}}}} \leqslant \frac{{{k_{{\text{at}}}}}}{{{k_{{\text{dyn}}}}}}N_{{{\text{cont}}}}^{2}.$$

In Eq. 4, the total number of dynamic atoms ${N_{{\text{dyn}}}}$ is expressed in terms of the dynamic mass and the atomic mass. It should, of course, be larger than or equal to the number of contacting atoms ${N_{{\text{cont}}}}$.

**Fig. 2:**
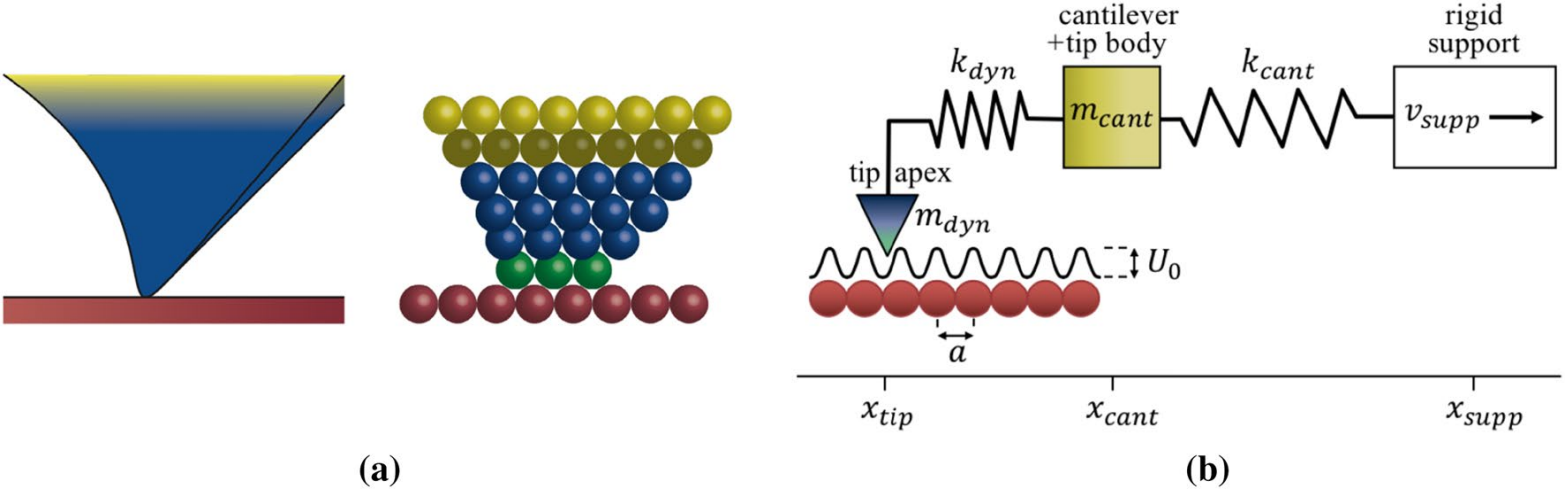
Schematic view of the deformation at the very tip apex, prior to a slip event. The green tip apex atoms in (**a**) are the ${N_{{\text{cont}}}}$ atoms that make contact with the red substrate. The blue atoms share most of the lateral displacement of the green atoms. Together with the green atoms, they establish the ${N_{{\text{dyn}}}}$ ‘dynamic’ atoms with mass ${m_{{\text{dyn}}}}$ that will be accelerated most strongly in the upcoming slip event. The lateral displacement of the yellow atoms is so modest, that these atoms are associated with the tip body, i.e., the rigid part of the tip. Panel (**b**) shows how this translates into a two-mass-two-spring model, illustrated here for the *x*-direction (see [3] and references therein)

Equation 4 provides us with a straightforward condition that needs to be satisfied in order to ‘see atoms’ in FFM-measurements. It is cast in the form of a relation between the number of tip atoms that are in direct contact with the substrate and the total number of atoms that can be considered to effectively move rigidly with these contact atoms, including the contact atoms themselves. In practice, both spring coefficients, ${k_{{\text{at}}}}$ and ${k_{{\text{dyn}}}}$, are in the order of 1 N/m, so that the ratio ${k_{{\text{at}}}}/{k_{{\text{dyn}}}}$ is approximately unity. This provides us with a unique way to estimate the typical dynamic mass in FFM-experiments, based on the mere fact that atomic patterns are often observed in these experiments, which implies that the inequality of Eq. 4 is often satisfied. For a typical, 10-atom contact (${N_{{\text{cont}}}}=10$), ${N_{{\text{dyn}}}}$ has to be below 100 atoms, corresponding to a maximum dynamic mass of approximately 10^−23^ kg. This is about 12 orders of magnitude lower than what is typically assumed, obviously satisfying the coarse, 10-orders-of-magnitude estimate that we arrived at above. The qualitative picture that goes with these small numbers is illustrated in Fig. 2, which shows the situation in which a significant fraction of the elastic deformation is concentrated in the very apex of the FFM-tip.

## Model Calculations

### General

The essential feature of our new description is formed by the high velocities to which the ultralow dynamic mass is accelerated, which enable efficient energy dissipation. We used numerical calculations for an extensive test of this scenario. To this end, we describe the combined system of the support, cantilever, flexible tip and small dynamic tip apex as a classical 2-mass-2-spring configuration [3, 27–30]. One mass is the low, dynamic mass ${m_{{\text{dyn}}}}$, associated with the very tip apex. It is connected via the effective spring ${k_{{\text{dyn}}}}$ to a large mass that corresponds to the rest of the tip and part of the cantilever. This large mass is, in turn, connected via the typical cantilever spring coefficient to the rigid support. As described in Appendix B, we employ a fully two-dimensional version of this model in the form of 2 × 2 coupled Langevin equations, each similar to Eq. 1. These are solved numerically, with special attention given to the enormous difference in the characteristic frequencies of the two pairs of coupled oscillators in this model. As in the real FFM-experiment, the displacement of the cantilever mass is monitored to construct the two-dimensional, lateral force maps.

### Numerical Results

**Fig. 3:**
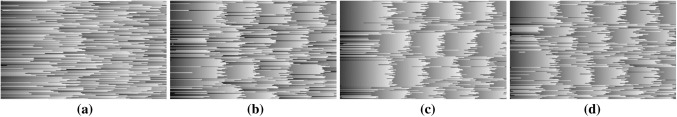
Numerically calculated lateral force maps, obtained by numerical integration of four coupled Langevin equations (see Appendix B), with the parameters chosen to match those of the FFM-experiment of Fig. 1 [4], namely ${k_{{\text{cant}}}}=30$ N/m, ${k_{{\text{dyn}}}}\,=\,2$ N/m, ${v_{{\text{supp}}}}=30$ µm/s, ${m_{{\text{cant}}}}={10^{ - 11}}$ kg, ${m_{{\text{at}}}}=74$ amu, ${U_0}=0.8$ eV (peak–peak), and $T=298$ K. The number of contacting atoms was set to ${N_{{\text{cont}}}}=100$ (see text). The number of dynamic atoms ${N_{{\text{dyn}}}}$ was chosen differently for each panel, in order to vary the damping regime. **a** ${N_{{\text{dyn}}}}={10^8},$ corresponding to one-hundredfold underdamped motion. **b** ${N_{{\text{dyn}}}}\,=\,{10^6},$ tenfold underdamped. **c** ${N_{{\text{dyn}}}}=4 \times {10^4},$ twofold underdamped. **d** ${N_{{\text{dyn}}}}=2.5\, \times \,{10^3},$ twofold overdamped. Note that the two underdamped cases in (**a**) and (**b**) show many multiple slip events and that the overdamped case shows an increase in the force fluctuations. For lateral force patterns with a well-defined lattice signature, the damping should be close to critical

Typical results of these model calculations are shown in Fig. 3. In order to keep calculation times manageable, we used a relatively large contact size of ${N_{{\text{cont}}}}\;=\,100$ atoms, corresponding to a damping rate of $\gamma \,=\,{10^{ - 10}}$ kg/s. Different values were chosen for ${N_{{\text{dyn}}}}$, in order to explore overdamped, critically damped and underdamped situations. The numerical results are in complete agreement with our qualitative description. Close to critical damping, more or less regular SS-motion is observed, which is reflected in patterns with clear atomic-scale periodicity.

Underdamping of the tip apex motion by only a few orders of magnitude (Fig. 3a) leads to a complete loss of atomic periodicity. As a result of the lack of damping, slip events frequently extend over multiple lattice spacings, which ruins the regularity.

Actually, the calculations show that also overdamping destroys the regular SS-signature in the FFM-patterns. This is caused by the fact that high damping goes hand in hand with a high level of thermal fluctuations, as a direct consequence of the fluctuation–dissipation theorem. Still, the loss of regular SS-patterns at overdamping may seem surprising. After all, at high damping all fluctuations are damped in a very short time. As a consequence, thermal activation of ‘early slips’ becomes ineffective, so that all slip events start very close to points of mechanical instability. In combination with the suppression of multiple slips, this might make one expect the SS-patterns to become very regular. However, such an expectation would be wrong. After all, the points of mechanical instability are determined by the force balance of Eq. 1 (or better, Eqs. 5 and 6) and the strong force fluctuations that accompany the strong damping introduce strong variations in the precise locations at which the slip events commence. This establishes the second mechanism by which the SS-patterns become more chaotic. This trend can be recognized in Fig. 3d.

Only in a limited window around critical damping, the dissipation rate is high enough to avoid frequent multiple slip events and the statistical force fluctuations are low enough to leave the pattern of slip positions well defined. As Eq. 4 indicates, calculations for other values of ${N_{{\text{cont}}}}$ give similar results, when ${N_{{\text{dyn}}}}$ is changed accordingly.

**Fig. 4:**
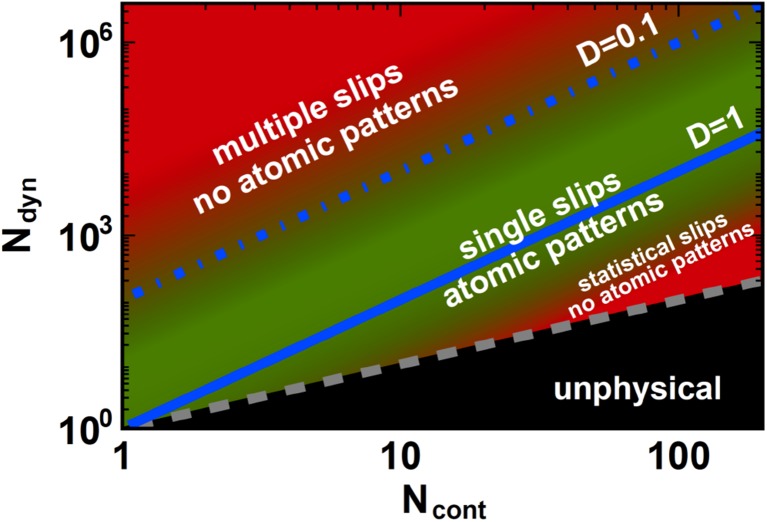
Friction ‘phase’ diagram as a function of the number of atoms ${N_{{\text{cont}}}}$ in the contact and the number of dynamic atoms ${N_{{\text{dyn}}}}$. Note that the accessible region in the diagram is that above the gray dashed line, ${N_{{\text{dyn}}}}\, \geqslant \,{N_{{\text{cont}}}}$. The blue line indicates the situation for critical damping of the tip apex motion, $~D \equiv {N_{{\text{cont}}}}/\sqrt {{N_{{\text{dyn}}}}} =1$. The colors indicate the quality of the stick-slip patterns. Green corresponds to a clearly recognizable atomic lattice, and red to strongly washed out patterns. Both underdamping and overdamping destroy the lattice signature, due to multiple slips and due to strong thermal fluctuations, respectively

Figure 4 summarizes our understanding in the form of a friction ‘phase’ diagram. Depending on the number of atoms in the contact ${N_{{\text{cont}}}}$ and the total number of atoms in the dynamic part of the tip ${N_{{\text{dyn}}}}$, the diagram goes from underdamped motion (upper left corner) with multiple slips, to overdamped behavior (lower right) with strong fluctuations. Only the intermediate regime around critical damping, $D\,=\,1$ in Fig. 4, is characterized by FFM-patterns with clear atomic periodicities. Note that ${N_{{\text{dyn}}}}$ cannot be lower than ${N_{{\text{cont}}}}$, so that the far lower right of the diagram is unphysical. For further details and trajectories of the tip and the cantilever, we refer to [31].

## Summary and Discussion

Stick-slip motion in friction force microscopy with clearly visible atomic periodicity is only possible by virtue of the high-speed motion that is carried out by a small and highly dynamic mass at the very end of the tip apex. The speed needs to be high enough to make the motion close to critically damped. Damping that would be either much higher or much lower would destroy the periodic patterns via excessively strong fluctuations or frequent slips of the tip over multiple lattice spacings. In this study, we arrived at this insight through estimates of the order of magnitude of the dissipative forces, contributed by the atoms in the contact and we formulated a condition on the effective shape of the tip apex, in Eq. 4. By integrating a set of four coupled Langevin equations, two for the *x*- and *y*-coordinates of the ‘regular’ tip-plus-cantilever combination and two for the *x*- and *y*-coordinates of the tiny dynamic mass at the end of the tip, we numerically explored the conditions that the tip needs to satisfy, which is summarized in the ‘phase’ diagram of Fig. 4.

The conclusion drawn here highlights a hitherto unrecognized signature of the flexibility and ultralow mass of the tip apex and the resulting, highly dynamic character of its motion. In several earlier publications [e.g., 3,27–30], we concentrated on cases where the atomic corrugation of the interaction potential between tip and substrate is relatively modest, so that the high rate, with which the rapidly moving tip is ‘attempting’ to overcome the energy barriers, can lead to a delocalization of the contact and a corresponding, extreme lowering of the friction force—an effect that we have nick-named “thermolubricity.” In the present manuscript, we focused on the increase in dissipation, associated with the high velocity that the tip acquires during a slip event, which is relevant for turning the sliding into nearly ideal atom-scale stick-slip motion, in case of a more pronounced corrugation of the interaction potential.

In view of the strict conditions found here for ${N_{{\text{cont}}}}$ and ${N_{{\text{dyn}}}}$, it may seem miraculous that many FFM-experiments yield force maps with clear, atomic-scale SS-motion. However, the tips used in AFM- and FFM-experiments have geometries that make them naturally fall in the central, green part of the diagram. In other words, even though we now understand that it is far from trivial that an FFM-experiment would be sensitive at all to the lattice periodicity, it is the typical shape of the employed tips that leads to a nearly critically damped motion of the tip apex, once it is brought in contact with the surface. We stress that the naïve, ideal FFM setup (Fig. 1c), equipped with a fully rigid tip apex, should always perform highly underdamped motion (upper left, far outside the diagram of Fig. 4) and should never produce atomic-scale stick-slip patterns.

What we have found here for a single asperity, moving over atomic distances, may also apply to frictional energy dissipation in practical contacts, consisting of large numbers of micrometer-scale contact regions. Both the geometry of each contact apex and the elasticity with which it is connected to the rest of the moving body determine the amount of energy each contact can dissipate. We speculate that when the connection between apex and body is made more rigid, e.g., by making the contacting surfaces nearly perfectly flat, the motion of the apices should become less strongly damped and the average friction force should reduce. The other extreme would be to pattern surfaces deliberately, for example in the form of nano-pillar arrays, to pre-define ${N_{{\text{dyn}}}}$ and ${k_{{\text{dyn}}}}$ in order to make the apex motion highly overdamped, which would maximize the friction force.

## Appendix A: Mechanism and Location of Dissipation: Arguments for Linear Scaling of Dissipation Rate with Contact Size

The main, but not sole mechanism (see below) of mechanical energy dissipation in friction force microscopy is related to the rapid motion of the tip apex with respect to the surface. It is accompanied by the creation of phonons in the substrate and in the tip. These phonons lead to the dissipation (irretrievable loss) of energy [19], and result (at a later stage) in thermalization. As mentioned in the main text, damping of the motion of an adsorbed atom on a surface is always close to critical. In the case of a sharp tip that is in contact with the surface via one single atom, phonons are produced not only in the substrate but also in the tip. As a result, the dissipation rate will then be higher than in the adsorbed-atom case by at most a factor two.

In this study, we have assumed the total dissipation rate, experienced when the tip apex is blunt, simply to be proportional to number of atoms in contact ${N_{{\text{cont}}}}$ (see Eq. 3). A justification for this can be given at more than one level. Obviously, if we consider each atom in the contact as a separate, independent (Einstein) oscillator that is vibrationally excited in the slip process and dissipates that vibrational energy independently, our model automatically exhibits the linear scaling of Eq. 3.

In a more realistic description, the ${N_{{\text{cont}}}}$ contacting atoms are treated as coupled oscillators, rather than independent ones. The total number of vibrational modes introduced by these atoms amounts to $3{N_{{\text{cont}}}} - 6$, which is nearly proportional to the contact size. If, on average, each of these modes contributes equally strongly to the energy dissipation, this description still results in near-linear scaling, very close to Eq. 3.

Of course, the vibrations that are relevant here are not limited to the $${N_{{\text{cont}}}}$$ contact atoms, but also involve nearby tip atoms that are not in contact with the counter-surface themselves. As a result, more than $$3{N_{{\text{cont}}}} - 6$$ modes will be involved and the effective number of modes contributing to the dissipation should scale super-linearly with $${N_{{\text{cont}}}},~$$for example approximately proportional to $$N_{{{\text{cont}}}}^{{{\raise0.7ex\hbox{$3$} \!\mathord{\left/ {\vphantom {3 2}}\right.\kern-0pt}\!\lower0.7ex\hbox{$2$}}}}$$ (under the assumption that the aspect ratios of the tip dimensions are all constant). Such an alternative scaling will lead to modifications in the powers of $${N_{{\text{cont}}}}$$ in Eqs. 3 and 4 (3/2 and 3, respectively, instead of 1 and 2) and a change in the horizontal axis of Fig. 4.

We should also mention the phonon discrimination mechanism that was proposed in [23] to explain surface diffusion of atomic clusters. Surface motion of a large (rigid) object can couple only to phonons in the substrate that have wavelengths that are comparable to or larger than the lateral size of the object. This discrimination effect actually leads to sublinear scaling with the size as $\sqrt {{N_{{\text{cont}}}}}$. Combining this sublinear tendency with the super-linear tendency mentioned in the previous paragraph, we may expect the naïve, linear scaling to provide a useful order-of-magnitude estimate.

We close this part by emphasizing that, in view of the low values of ${N_{{\text{cont}}}}$, considered here, the numerical effects of any non-linearity will necessarily be modest and the qualitative conclusions reached in this article should remain valid.

For completeness, also more ‘remote’ channels of mechanical energy dissipation may be active in friction force microscopy. For example, the cantilever may exhibit internal damping. Since the quality factors measured for free cantilevers are typically in the order of thousands [14], this internal damping must be negligibly weak. Similarly, the internal damping of the bending motion of the tip or its apex must be insignificant, since the quality factors measured for cantilevers with the tip in contact with a substrate are still relatively high, in the order of hundreds [14]. These observations justify the choice in our calculations not to introduce explicit damping in Eqs. 7 and 8 (see Appendix B), and not to include terms depending on the relative velocity of the tip apex and the cantilever in Eqs. 5 and 6. Note that Eqs. 5–8 do imply modest, indirect damping of the cantilever via the slow, forced motion of the tip with respect to the substrate; this effect is smaller than the explicit damping of the rapid mode by a factor ${m_{{\text{dyn}}}}/{m_{{\text{cant}}}}$.

## Appendix B: Details of the Numerical Calculations

### Equations of Motion

In our calculations, we numerically integrated the equations of motion for the dynamic tip mass, ${m_{{\text{dyn}}}},$ and of the combination of the rest of the tip and the moving part of the cantilever, ${m_{{\text{cant}}}}.$ Both were followed in two dimensions, leading to the following four coupled Langevin equations:

5 $${m_{{\text{dyn}}}}{\ddot {x}_{{\text{tip}}}}= - {\left. {\frac{{\partial {V_{{\text{int}}}}\left( {x,y} \right)}}{{\partial x}}} \right|_{\left( {x,y} \right)=\left( {{x_{{\text{tip}}}},{y_{{\text{tip}}}}} \right)}} - {k_{{\text{dyn}}}}\left( {{x_{{\text{tip}}}} - {x_{{\text{cant}}}}} \right)+{\xi _x} - \gamma {\dot {x}_{{\text{tip}}}},$$

6 $${m_{{\text{dyn}}}}{\ddot {y}_{{\text{tip}}}}= - {\left. {\frac{{\partial {V_{{\text{int}}}}\left( {x,y} \right)}}{{\partial y}}} \right|_{\left( {x,y} \right)=\left( {{x_{{\text{tip}}}},{y_{{\text{tip}}}}} \right)}} - {k_{dyn}}\left( {{y_{{\text{tip}}}} - {y_{{\text{cant}}}}} \right)+{\xi _y} - \gamma {\dot {y}_{{\text{tip}}}},$$

7 $${m_{{\text{cant}}}}{\ddot {x}_{{\text{cant}}}}= - {k_{{\text{cant}}}}\left( {{x_{{\text{cant}}}} - {x_{{\text{supp}}}}} \right) - {k_{{\text{dyn}}}}\left( {{x_{{\text{cant}}}} - {x_{{\text{tip}}}}} \right),$$

8 $${m_{{\text{cant}}}}{\ddot {y}_{{\text{cant}}}}= - {k_{{\text{cant}}}}\left( {{y_{{\text{cant}}}} - {y_{{\text{supp}}}}} \right) - {k_{{\text{dyn}}}}\left( {{y_{{\text{cant}}}} - {y_{{\text{tip}}}}} \right).$$

Note that Eqs. 7 and 8 have no damping and no noise term, which reduces them to straightforward Newton equations of motion. In our calculation, damping and noise exclusively derive from the tip–surface contact and are therefore only explicitly present in Eqs. 5 and 6. Here, the spring coefficients have been chosen equal for the *x-* and *y-*directions, but they can be replaced easily by an anisotropic choice. In our computational scheme, we numerically integrated these equations, using the Verlet algorithm to periodically update the four velocities, ${\dot {x}_{{\text{tip}}}},$ ${\dot {y}_{{\text{tip}}}}$, ${\dot {x}_{{\text{cant}}}},$ and ${\dot {y}_{{\text{cant}}}},$ and the four positions, ${x_{{\text{tip}}}},$ ${y_{{\text{tip}}}},$ ${x_{{\text{cant}}}},$ and ${y_{{\text{cant}}}},~$iterating with a time step ${\text{d}}t$ [32].^1^

### Interaction Potential

The two-dimensional periodic potential ${V_{{\text{int}}}}$ describes the interaction between the tip apex and the crystal surface over which the tip is forced to slide. Here, we used a potential with the symmetry and lattice period of graphite, based on the potential that was used before by Verhoeven et al. [33 and references therein].

9 $${V_{{\text{int}}}}\left( {x,y} \right)= - \frac{2}{9}{U_0}\left[ {2{\text{cos}}\left( {{a_1}x} \right){\text{cos}}\left( {{a_2}y} \right)+{\text{cos}}\left( {2{a_2}y} \right)} \right]$$

with ${a_1}=2\pi /\left( {0.246~{\text{nm}}} \right)$ and ${a_2}=2\pi /\left( {0.426~{\text{nm}}} \right)$, determined by the periodicity of the graphite surface. The prefactor of $2/9$ serves to make the peak-to-peak variation of ${V_{{\text{int}}}}$ equal to ${U_0}.$

### Time Step

For the time step ${\text{d}}t$ between subsequent iterations, we introduce two upper limits. On the one hand, ${\text{d}}t$ should be short enough to capture the behavior of the most dynamic element in the system, namely the dynamic tip. Therefore, we respect one upper limit for ${\text{d}}t$ as 10% of the vibrational period of the tip apex,

10 $${\text{d}}t_{1}^{{max}}=0.1 \cdot 2\pi \sqrt {\frac{{{m_{{\text{dyn}}}}}}{{{k_{{\text{dyn}}}}+{k_{{\text{latt}}}}}}},$$

in which ${k_{{\text{latt}}}}$ is the maximum force derivative by which the interaction potential contributes to the total spring constant, experienced by the tip apex.

The second constraint on the time step derives from the random force, generated via the noise terms ${\xi _x}$ and ${\xi _y}$ in Eqs. 5 and 6. In case of strong dissipation, the noise will be high too, as they are related via the fluctuation–dissipation theorem. Hence, the influence of the noise on the acceleration will be high and thus we need to choose an accordingly small time step. The characteristic timescale of the noise is set by the dissipation rate and the mass, and we verified that our numerical results are sufficiently reliable and stable when we keep ${\text{d}}t$ below 20% of this time, which defines our second upper limit as

11 $${\text{d}}t_{2}^{{{\text{ma}}x}}=0.2 \cdot \frac{{{m_{{\text{dyn}}}}}}{\gamma }.$$

We combine these two conditions into $dt={\text{min}}\left( {dt_{1}^{{{\text{max}}}},dt_{2}^{{{\text{max}}}}} \right).$

### Noise Term

The noise term $\xi$ in Eqs. 5 and 6 is provided in the calculations as a Gaussian-distributed, uncorrelated random number with a standard deviation in accordance with the fluctuation–dissipation theorem $\left< {\xi \left( t \right)\xi \left( {t'} \right)}\right>=2\gamma {k_B}T\delta \left( {t - t'} \right)$, where ${k_B}$ is the Boltzmann constant. Actual $\xi$-values are generated in our calculations by means of regular, uniformly distributed random numbers and a Box–Muller transformation [34], in order to obtain a Gaussian distribution. We verified that this procedure indeed generates a Gaussian distribution of random forces, with an average of zero and a mean square value satisfying the fluctuation–dissipation theorem for the specific combination of temperature $T$ and dissipation rate $\gamma$ of the calculation.

### Damping of the Cantilever

In the numerical calculations reported, no damping was applied to the cantilever. As typical cantilevers have very high-quality factors (thousands), even in contact (hundreds) [14], both the damping and the noise on the cantilever motion are relatively small (see also Appendix A). Calculations were performed to check this in case of a close-to-critically-damped tip apex and almost no effect on the dynamic tip apex behavior was observed at typical cantilever damping values. Taking confidence from these observations, we set the damping of the cantilever to zero in all calculations reported here, which resulted in a significant reduction in calculation time.

### Scan Trajectory of the Support

In FFM-experiments, like in most scanning probe experiments, the support is scanned over the surface in a line-by-line fashion. In our calculations, we oriented our coordinate system such that the scan lines were oriented along the *x*-axis. Every combination of a forward and subsequent backward line was performed at a fixed *y*-coordinate of the support. Prior to the next forward–backward line pair, the support made a small step along the *y*-direction.

We have faithfully followed this scan sequence in our calculations, because it leads to a characteristic hysteresis in the lateral force maps, both along the *x*-direction and, after a few initial scan lines, also along the *y*-direction. For example, during the initial part of each forward line, the *x*-force first has to reduce to zero, change sign, and build up in the opposite direction to a level high enough to induce slip events. Such hysteresis is commonly observed in experiments [2] and is reproduced well by our calculations, indicating that not only the typical slip-induced force variations in the calculations are similar to those in experiments, but also the typical force level at which slip events take place.
